# Comprehensive analysis of mutations in the *MEFV* gene reveal that the location and not the substitution type determines symptom severity in FMF

**DOI:** 10.1002/mgg3.336

**Published:** 2017-10-09

**Authors:** Mike M. Moradian, Davit Babikyan, Dion Banoian, Hasmik Hayrapetyan, Hakob Manvelyan, Nareh Avanesian, Tamara Sarkisian

**Affiliations:** ^1^ Department of Molecular Genetics Morava Scientific & Technology Services Glendale California; ^2^ Department of Molecular Genetics Center of Medical Genetics and Primary Health Care Yerevan Armenia; ^3^ Department of Clinical Genetics Yerevan State medical University Yerevan Armenia

**Keywords:** Allele frequency, amino acid substitution, clustering, conserved, FMF, *MEFV*, mutation, nonsynonymous, pyrin domain, radical/conservative ratio

## Abstract

**Background:**

Familial Mediterranean Fever (FMF) is an autoinflammatory disorder caused by mutations in the *MEFV* gene. These mutations appear in different populations with different frequencies and their caused symptom severities vary from mild to moderate to severe depending on the mutation type.

**Methods:**

In this study, we analyzed the mutations that have been reported in the *MEFV* gene from symptomatic FMF patients and compared their frequencies in different populations from the 1000 Genome and the Exome databases, using statistical clustering. We also analyzed the nucleotide and amino acid substitution patterns across the *MEFV* gene.

**Results:**

We found 16 (8%) nonsynonymous mutations outside exon 10 that did not cluster with known disease‐causing mutations (DCMs), due to their high frequencies in other populations. We also studied the substitution patterns for nucleotides and amino acids to determine the conserved and variable regions in the *MEFV* gene. In general more nonsynonymous substitutions were reported in exons 2, 3, and 10 from the FMF database (symptomatic FMF patients) compared to the 1000 Genome and the Exome databases. The same was true for amino acid (AA) substitutions where there were 1.5 times more radical (RAD) to conservative (CON) changes. However, when it came to AA substitutions exon 10 was quite conserved with a RAD/CON ratio of 0.9. In fact, we report that the most severe FMF symptoms are caused by conservative mutations in two highly conserved exon 10 regions.

**Conclusion:**

We found presumptive FMF‐causing mutations that did not cluster with DCMs based on their allele frequencies. We also observed that the type of mutation is less likely to determine the severity of the FMF symptoms; rather it was the location of the mutations that was the determining factor.

## Introduction

Familial Mediterranean Fever (FMF, OMIM 249100) is an autoinflammatory disorder characterized by recurrent episodes of fever, peritonitis, pleuritis, synovitis, and complications of amyloidosis (Sohar et al. 1967; French FMF Consortium. 1997). FMF is considered the prototype among the hereditary recurrent fevers as the most frequent and the first whose gene has been identified (Heller et al. 1955).

This autosomal recessive condition is usually caused by mutations in the *MEFV* gene (OMIM 608107; GenBank NM_000243.2), which has 10 exons and codes for a protein called pyrin (French FMF Consortium 1997; International FMF Consortium 1997). More than 300 mutations in the *MEFV* gene, found in symptomatic FMF patients, have been reported in the FMF mutation database, Infevers, a database dedicated to auto‐inflammatory mutations (Milhavet et al. 2008; Touitou et al. 2014). Although FMF mutations have been reported in every exon, the mutations in exon 10 of the *MEFV* gene are mostly responsible for causing FMF symptoms (Booth et al. 1998; Aksentijevich et al. 1999). Weinert's group identified a group of 28 disease‐causing mutations (DCMs) and their impact on the protein structure of pyrin (Weinert et al. 2009). Thus these mutations were designated as reference mutations for clustering analysis in this study and referred to as DCMs.

Mutation frequencies in different populations have been used to indicate if the carrier frequencies are different among populations. In fact, many bioinformatics pipelines include mutation allele frequencies from the 1000 Genome Project database (Abecasis et al. 2012) for comparisons. A statistical clustering analysis using allele frequencies in different population could reveal more detail information. This is particularly true for diseases such as FMF that are more prevalent in certain geographic areas, countries, and ethnic populations. Comparison between the allele frequencies from known DCMs from populations with FMF and others could provide additional information for the analysis of all mutations and their contributions to the diseases.

In this study we performed a comprehensive mutation analysis, including molecular evolution analysis, for 310 mutations reported in the FMF database. We studied the substitution patterns for both nucleotide and amino acid changes for all nonsynonymous mutations and identified the nonsynonymous to synonymous (d*n*/d*s*) and the radical to conservative (RAD/CON) ratios for amino acid substitutions. We also used the allele frequencies of mutations in populations reported in the 1000 Genome Project (Abecasis et al. 2012) and the Exome Project (Lek et al. 2016) to conduct a clustering analysis with known DCMs. We used the results from these analyses to identify the areas in the *MEFV* gene and the pyrin protein that are less conserved and allow more variation versus more conserved and less variable regions. We also identified the mutations that did not cluster with DCMs based on their allele frequencies.

## Materials and Methods

### Ethical compliance

This study was approved by the ethics committee of the Yerevan State Medical University.

### Databases and mutation analysis

The mutations were downloaded from “The Registry of Hereditary Auto‐inflammatory Disorders Mutations” (Milhavet et al. 2008; Touitou et al. 2014), the 1000 Genome project (Abecasis et al. 2012), and the Exome database (Lek et al. 2016). The populations genetics information for the mutations were downloaded from the 1000 genome database and the Exome database individually, concatenated in an excel file, and formatted for cluster analysis. The clustering analysis was performed using the open source clustering software, Gene Cluster 3.0 Program (De Hoon et al. 2004). We used the k‐means clustering algorithm for this analysis with the following settings: organized genes, five clusters, 100 runs, and Euclidean distance as similarity metric. We checked the results against a hierarchical clustering algorithm and got similar groupings. We used five clusters since using more clusters just split the smaller clusters with no real impact on the groupings with the DCMs. The SIFT (Ng and Henikoff 2003) (http://sift.jcvi.org/www/SIFT_enst_submit.html) and PolyPhen (Adzhubei et al. 2010) (http://genetics.bwh.harvard.edu/pph2/) results were from the 1000 Genome database result download and were confirmed independently. The analyzed mutations were as follows: p.V33L, c.97G>T, rs11466016; p.R42W, c.124C>T, rs61754767; p.L110P, c.329T>C, rs11466018; p.E148Q, c.442G>C, rs3743930; p.E167D, c.501G>C, rs104895079; p.G196W, c.586G>T, rs104895179; p.R202Q, c.605G>A, rs224222; p.E230K, c.688G>A, rs104895080; p.S242R, c.726C>G, rs104895178, c.726C>A, rs104895197; p.G304R, c.910G>A, rs75977701; p.A311V, c.932C>T, rs74346519; p.R329H, c.353G>A, rs104895112; p.P369S, c.459G>T, rs11466023; and p.R408Q, c.583G>A, rs11466024; p.Q440E, c.652C>T, rs11466026; p.R461Q, c.737C>T, rs145637617; p.F479L, c.1437C>G, rs104895083; p.V487L, c.825C>T, rs104895100; p.I591T, c.1772T>C, rs11466045; p.M680I, rs28940580, c.2040G>C; p.M694V,rs61752717, c.2080A>G; p.V726A rs28940579, c.2177T>C.

### Conservation study and species information

The nonsynonymous to synonymous (d*n*/d*s*) and the radical to conservative (RAD/CON) ratios were calculated manually. The type of mutation was counted in every database and the ratio was obtained by simple division of the corresponding values. The *MEFV* nucleotide and protein sequence analysis of these ratios were performed on the following species: *Homo Sapiens* (Human) Chromosome 16, NC_000016.10 (3242028–3256776, complement), nucleotide (NM_000243.2), protein (NP_000234.1); *Pan troglodytes* (chimpanzee) Chromosome 16, NC_006483.4 (3505969–3520855, complement), nucleotide (XM_016929284.1), protein (XP_003815187.1); *Macaca mulatta* (Rhesus monkey) Chromosome 20, NC_027912.1 (3678290–3693084), nucleotide (XM_001092338.3), protein (XP_001092338.2); *Papio anubis* (olive baboon) Chromosome 20, NC_018171.2 (3049027–3063645, complement), nucleotide (XM_017953124.2), protein (XP_003916489.1); *Gorilla gorilla* (western gorilla) Chromosome16, NC_018440.2 (3375831–3390521, complement), nucleotide (XM_004057075.2), protein (XP_004057123.1); *Pongo abelii* (Sumatran orangutan) Chromosome16 NC_012607.1 (3354206–3368798, complement), nucleotide (XM_003778553.2), protein (XP_003778601.1). The nucleotide and amino acid sequences were aligned using Clustal X program (Higgins and Sharp 1988; Larkin et al. 2007) and the ratios were calculated manually.

### Sequencing of healthy population

The sequence analysis of healthy Armenian individuals was performed on peripheral whole blood samples collected in EDTA tubes. All 10 exons and up to 100 base pairs of intronic regions were amplified using M13‐tailed oligonucleotide primers and PCR conditions described previously (International FMF Consortium 1997; Aksentijevich et al. 1999). The amplified DNA from all exons was sequenced at the National Institute of Health, Bethesda, Maryland, USA in 2001, using an Applied Biosystems (ABI) 377 sequence analyzer from Thermo Fisher Scientific, (Waltham, Massachusetts). The generated DNA sequences were analyzed using Sequencher software 3.0 Gene Codes Corporation, (Ann Harbor, MI).

## Results

### FMF mutation database information

We reviewed 310 reported mutations in the FMF mutation database; they were 217 nonsynonymous, 55 synonymous, 25 intronic, nine 5′ flanking region, one 5′ UTR, and three 3′ UTR. 202 of these mutations were submitted to the database through personal communications, and the other 108 mutations were mentioned in either publications or Abstracts. Several of these mutations were designated as pathogenic through genotype/phenotype correlations and the presence of these mutations in symptomatic FMF patients. However, three of these mutations, (p.M680I, rs28940580, c.2040G>C; p.M694V, rs61752717, c.2080A>G; p.V726A rs28940579, c.2177T>C), have been proven to cause FMF and have the highest frequency of occurrence among FMF patients. Therefore, we used these three known FMF‐causing mutations as reference mutations and refer to them as DCMs.

### Study of molecular evolution parameters in the *MEFV* gene

We analyzed the nucleotide substitution patterns for mutations reported in the FMF, 1000 Genome, and Exome databases (hereafter the three databases). The synonymous substitutions were present in exon 1 (3), exon 2 (23), exon 3 (6), exon 5 (7), exon 9 (2), and exon 10 (15). Exon 2 and exon 10 had higher synonymous substitutions when normalized as based on their size compared to the other exons.

The numbers of nonsynonymous substitutions were 202, 248, and 117 in the FMF, 1000 Genome, and Exome databases, respectively. We calculated the nonsynonymous to synonymous (d*n*/d*s*) ratios, for exons 1, 2, 3, 5, and 10. We excluded exons 4, 6, 7, 8, and 9 since the substitution ratios could be misleading due to their short length. We compared the d*n*/d*s* ratios for substitutions reported in all three databases. In the FMF database, which houses the mutations reported from symptomatic population, the d*n*/d*s* ratio had mix results. The d*n*/d*s* ratios for exons 2, 3, and 10 were higher in the FMF database compared to the 1000 genome and Exome databases (*P* value 0.05). This was expected since the data reported to the FMF database came from symptomatic individuals who were more likely to have nonsynonymous substitutions (Table 1). Exome database had the lowest d*n*/d*s* ratios, perhaps due to its massive data size; the only exception was exon 1.

**Table 1:** Nonsynonymous to synonymous (d*n*/d*s*) ratios

	d*n*/d*s*		
	FMF	1000	Exome
Exon 1	2.33	2.23	3.40
Exon 2	3.41	2.37	1.36
Exon 3	3.83	2.35	1.45
Exon 5	2.43	3.27	1.29
Exon 10	4.73	3.21	2.22

Nonsynonymous to synonymous (d*n*/d*s*) ratios calculated from the FMF, the 1000 Genome, and the Exome database.

### Amino acid substitutions

Analysis of the amino acid substitutions from the FMF database revealed more radical substitutions (120) than conservative substitutions (82), almost 1.5 times more. Interestingly, the three DCMs responsible for severe FMF symptoms (i.e., p.M680I, p.M694V, and p.V726A) were conservative changes with benign or tolerable SIFT and PolyPhen designations. We realized that this could be dependent on the location of the substitution in the protein where changes in the functional parts of the protein could be less tolerated than changes in the nonfunctional areas. Looking at the details of substitutions in exons from the FMF database we determined that exon 10 had the lowest number of radical to conservative substitution ratio, indicating more conservation for this exon. On the other hand, exon 2 had the highest number of radical substitutions (Table 2), suggesting less conservation. Exons 3 and 5 are relatively conserved with an equal number of radical and conservative substitutions. Analysis of substitutions in exon 1 in the FMF database generated unusual and inconsistent results since it had five times more radical substitutions, perhaps due to a low number of substitutions (i.e., 6). The Same study conducted on the Exome database generated similar patterns yet lower amino acid substitution ratios. These ratios were lower since they represent a large dataset from random population with regards to *MEFV* substitutions. Analysis of data from 1000 Genome database was also similar in resulted patterns, indicating less conservation in exon 2 compared to the other exons and most conservation in exon 10. We also identified two areas with high number of conservative substitutions in exon 10. The first segment was between amino acid 680 to 704 with a RAD/CON ratio of 0.5 (4/8); the second area was between amino acid 717 and 743 with no radical substitutions compared to 9 conservative ones. These two areas presumably represent conserved areas analyzed in more details in the discussion section. Conversely, exon 2 had areas with high radical substitution and consequently higher RAD/CON ratios. The First area was between amino acid 136 to 166 with a RAD/CON ratio of 6 (12/2). The second area was between amino acids 266 to 304 with a RAD/CON ratio of almost 4 (11/3). One small area of high radical substitutions in exon 2 was between amino acids 222 to 232 with RAD/CON ratio of 6 to 1.

**Table 2:** Radical (RAD) to conservative (CON) amino acid ratios

	Radical substitutions			Conservative substitutions			RAD/CON ratio		Exome
	FMF	1000	Exome	FMF	1000	Exome	FMF	1000	
Exon 1	5	14	4	1	11	9	5.0	1.3	0.4
Exon 2	50	42	25	20	14	19	2.5	3.0	1.3
Exon 3	12	22	9	11	16	8	1.1	1.4	1.1
Exon 5	8	15	2	8	15	7	1.0	1.0	0.3
Exon 10	32	33	9	35	37	12	0.9	0.9	0.8

Radical (RAD) to conservative (CON) amino acid ratios calculated from the FMF, the 1000 Genome, and the Exome databases.

We also identified the highest number of amino acid substitution types to be: IV(8), RH(8), EK(7), AV(6), ED(6), GR(6), LP(6), RC(5), SC(5), TI(5), AT(4), PL(4), PR(4), SN(4), presenting a mixed pattern with regards to the type of the amino acid substitution.

### Interspecies comparison of pyrin protein conservation

We conducted an interspecies comparison for d*n*/d*s* and RAD/CON ratios to study the pyrin protein conservation between different species. We chose to perform these analyses on primates only since the distant species had highly divergent sequences with some missing an exon or two. Our analysis showed that exons 1,3, and 5 were fairly conserved in primates with lower rates of substitutions therefore we focused on exons 2 and 10, which represent two important functional domains of the protein. On the nucleotide level all d*n*/d*s* ratios showed higher conservation for exon 10 compared to exon 2, except in orangutan. Not surprisingly, this conservation was more distinct between more closely relates species, that is, chimpanzee and gorilla (Table 3). On the amino acid level three species did not have any conservative substitutions in exon 10, therefore we could not calculate RAD/CON ratios. Yet, the number of radical substitutions in exon 2 were much higher in these species, indicating tolerance for radical substitutions. In the other two species, rhesus monkey and baboon, the RAD/CON ratios were closer for exon 2 and exon 10, yet similarly the number of radical substitutions were much higher in exon 2 (Table 3). The interspecies comparison also supported that exon 2 is relatively less conserved than exon 10 in the *MEFV* gene.

**Table 3:** Interspecies analysis of d*n*/d*s* and RAD/CON between primates & human

	Chimpanzee	Gorilla	Orangutan	Rhesus monkey	Baboon
	d*n*/d*s* = ratio				
Exon2	8/5 = 1.6	10/5 = 2	24/12 = 2	32/10 = 3.2	34/11 = 3.1
Exon 10	1/4 = 0.5	1/9 = 0.1	12/6 = 2	11/13 = 0.8	21/14 = 0.9
	RAD/CON = ratio				
Exon2	4/2 = 2	4/2 = 2	10/5 = 2	18/9 = 2	27/13 = 2.1
Exon 10	1/0	1/0	6/0	6/4 = 1.5	10/5 = 2

Analysis of nonsynonymous to synonymous (d*n*/d*s*) and radian to conservative (RAD/CON) ratios for exons 2 and 10 between human (*Homo Sapiens* (Human) Chromosome 16, NC_000016.10 (3242028..3256776, complement), nucleotide (NM_000243.2), protein (NP_000234.1); and five primates (*Pan troglodytes* (chimpanzee) Chromosome 16, NC_006483.4 (3505969..3520855, complement), nucleotide (XM_016929284.1), protein (XP_003815187.1); *Macaca mulatta* (Rhesus monkey) Chromosome 20, NC_027912.1 (3678290..3693084), nucleotide (XM_001092338.3), protein (XP_001092338.2); *Papio anubis* (olive baboon) Chromosome 20, NC_018171.2 (3049027..3063645, complement), nucleotide (XM_017953124.2), protein (XP_003916489.1); *Gorilla gorilla* (western gorilla) Chromosome16, NC_018440.2 (3375831..3390521, complement), nucleotide (XM_004057075.2), protein (XP_004057123.1); *Pongo abelii* (Sumatran orangutan) Chromosome16 NC_012607.1 (3354206..3368798, complement), nucleotide (XM_003778553.2), protein (XP_003778601.1). Exon 10 for chimpanzee, gorilla, and orangutan had no radical amino acid substitutions therefore no ratio.

### Healthy carrier mutation frequencies

The carrier mutation frequency in 286 healthy Armenian individuals was determined using full gene sequencing. We compared the carrier allele frequency of 15 mutations present in healthy Armenian population with those of five major populations from 1000 Genome and Exome projects. We found significant differences in allele frequencies between Armenians and the other populations for mutations found in exons 5 and 10 (*P* value <0.05 for). Yet when all mutations were considered, only the African population had significant differences (*P* value = 0.006); all the other populations had somewhat similar allele frequencies. In fact, in some cases the incidence of the mutation was higher, most notably, about 6 times higher incidence of p.E148Q (c.442G>C, rs3743930) mutation in East and South Asian populations (Table 4). Although the incidence of the 3 DCMs, that is, p.M680I, p.M694V, and p.V726A in the Armenian population were 3%, 4%, and 6%, respectively, they were almost absent from all the other populations’ healthy carriers. Similarly, the incidents of p.F479L (c.1437C>G, rs104895083) and p.E167D(c.501G>C, rs104895079) mutations, which are considered DCMs in the Armenian population were 0.7% and were absent from the other populations. In general, the mutations that could cause moderate to severe FMF symptoms, which were present in the healthy carrier Armenian population, were absent in healthy individuals from the other populations.

**Table 4:** The carrier mutation frequency in 286 healthy Armenian individuals

Mutation	Armenian	African		American^a^		East‐Asian		South‐Asian		European^a^	
		1000 Genome	Exome	1000 Genome	Exome	1000 Genome	Exome	1000 Genome	Exome	1000 Genome	Exome
p.R42W	0.000	0.008	0.004	0	0.000	0.01	0.000	0.005	0.000	0	0.000
p.E148Q	0.052	0.020	0.018	0.012	0.022	0.289	0.315	0.305	0.302	0.009	0.020
p.E167D	0.007	NA	0.000	NA	0.000	NA	0.000	NA	0.000	NA	0.000
p.R202Q	0.180	0.036	0.079	0.324	0.413	0.031	0.047	0.099	0.108	0.278	0.304
p.E230K	0.000	0.000	0.000	0.000	0.000	0.000	0.000	0.006	0.004	0.001	0.000
p.T267I	0.000	0.000	0.000	0.000	0.000	0.000	0.000	0.001	0.000	0.000	0.000
p.P369S	0.017	0.002	0.004	0.007	0.005	0.067	0.072	0.021	0.015	0.004	0.010
p.R408Q	0.014	0.002	0.004	0.007	0.004	0.055	0.054	0.02	0.014	0.004	0.010
p.F479L	0.007	NA	0.000	NA	0.000	NA	0.000	NA	0.000	NA	0.000
p.M680I	0.028	NA	0.000	NA	0.000	NA	0.000	NA	0.000	NA	0.000
p.M694V	0.042	0	0.000	0.001	0.000	0	0.000	0	0.000	0	0.000
p.M694I	0.004	NA	0.000	NA	0.001	NA	0.000	NA	0.000	NA	0.000
p.K695R	0.004	0	0.000	0.003	0.002	0	0.000	0	0.000	0.007	0.008
p.V726A	0.056	0	0.000	0	0.000	0	0.000	0	0.000	0.001	0.003
p.A744S	0.007	0	0.000	0.006	0.002	0	0.000	0	0.000	0.005	0.002
p.R761H	0.007	NA	0.000	NA	0.000	NA	0.002	NA	0.000	NA	0.000

NA = no population frequency available from the 1000 Genome, perhaps since none of the sequenced populations had this mutation.

a Depicts populations with possible MEFV mutation carriers.

### Cluster analysis of mutation frequencies

We performed a clustering statistical analysis using frequency of mutations in the *MEFV* gene in all five major populations and 26 subpopulations from the 1000 Genome Project. The goal was to identify the mutations that do not cluster with the known DCMs based on their frequencies in populations prone to FMF and otherwise (Table 5). We identified 16 mutations that did not cluster with the DCMs (*P*‐value <0.05). These mutations were in the following exons: 2 in exon 1 (p.V33L, c.97G>T, rs11466016; p.R42W, c.124C>T, rs61754767), 6 in exon 2 (i.e., p.L110P, c.329T>C, rs11466018; p.E148Q, c.442G>C, rs3743930; p.G196W, c.586G>T, rs104895179; p.R202Q, c.605G>A, rs224222; p.E230K, c.688G>A, rs104895080; p.G304R, c.910G>A, rs75977701), 4 in exon 3 (p.A311V, c.932C>T, rs74346519; p.R329H, c.353G>A, rs104895112; p.P369S, c.459G>T, rs11466023; p.R408Q, c.583G>A, rs11466024), 1 in exon 4 (p.Q440E, c.652C>T, rs11466026), 2 in exon 5 (p.R461Q, c.737C>T, rs145637617; p.V487L, c.825C>T, rs104895100), and 1 in exon 9 (p.I591T, c.1772T>C, rs11466045). These mutations had significant allele frequency differences with DCMs and did not cluster with them in analyzed populations therefore they were designated as nonclustered mutations. Clustering analysis was also performed using the wild type and heterozygous genotype frequencies in the same populations, which generated similar results to when allele frequencies were used. The same analysis was performed on mutations identified in the Exome Project using allele frequencies in the seven major populations. In this case we found six mutations that had significant allele frequency differences with the DCMs and thus did not cluster with them. They were p.E148Q and p.R202Q in exon 2, p.P369S and p.R408Q in exon 3, p.G436R in exon 4, and p.I591T in exon 9. Although the number of sequences reported in the Exome database is several times that of the sequences reported in the 1000 Genome, the number of nonclustered mutations with the DCMs was less (i.e., 16 vs. 6).

**Table 5:** Clustering statistical analysis using frequency of mutations in the MEFV gene in all five major populations and 26 subpopulations from the 1000 genome project

Mutation	ALL	AFRICAN								AMERICAS					EAST ASIAN						EUROPEAN						SOUTH ASIAN						*P*‐value
		AFR	ACB	ASW	ESN	GWD	LWK	MSL	YRI	AMR	CLM	MXL	PEL	PUR	EAS	CDX	CHB	CHS	JPT	KHV	EUR	CEU	FIN	GBR	IBS	TSI	SAS	BEB	GIH	ITU	PJL	STU	
p.V33L	0.2	0.6	1	0.8	0.4	0.4	0	1.8	0.5	0.1	0	0	0	0.1	0	0	0	0	0	0	0	0	0	0	0	0	0	0	0	0	0	0	0.006
p.R42W	0.2	0.8	1	0	1	0.4	1	0.6	0.9	0	0	0	0	0	0	0	0	0	0	0	0	0	0	0	0	0	0	0	0	0	0	0	0.003
p.L110P	1.2	0	0	0	0	0	0	0	0	0	0	0	0	0	5.9	2.7	7.8	7.1	5.8	5.6	0	0	0	0	0	0	0	0	0	0	0	0	0.006
p.E148Q	12.6	2	1	3.3	1.5	0.4	7.1	0.6	0.9	1.2	0	1.6	1.2	1.9	28.9	32.8	28.6	29	21.6	32.8	0.9	1	0	2.7	0	0.9	30.5	34.3	42.7	26	26	23.5	0.000
p.G196W	0.6	1.9	2.6	0	3	1.3	0.5	1.2	3.7	0.4	0	0	0.6	1	0	0	0	0	0	0	0	0	0	0	0	0	0	0	0	0	0	0	0.002
p.R202Q	13.6	3.6	3.1	11.5	2	4.4	2.5	2.9	1.9	32.4	28.7	37.5	40	26.4	3.1	4.3	2.4	4.8	1.9	2	27.8	24.7	33.8	22	29.4	28.5	9.9	9.3	12.6	9.8	8.3	9.3	0.000
p.230EQ	0.1	0	0	0	0	0	0	0	0	0	0	0	0	0	0	0	0	0	0	0	0.1	0.5	0	0	0	0	0.6	1.7	0	1.5	0	0	0.030
p.G304R	0.7	0	0	0	0	0	0	0	0	0	0	0	0	0	3	5.4	2.4	1.9	2.9	2.5	0.7	0	3.5	0	0	0	0	0	0	0	0	0	0.003
p.A311V	0.1	0.3	0	0	1	0	0	0	0.9	0	0	0	0	0	0	0	0	0	0	0	0	0	0	0	0	0	0	0	0	0	0	0	0.048
p.R329H	0.2	0	0	0	0	0	0	0	0	0.1	0.5	0	0	0	0	0	0	0	0	0	0.2	0	0	0	0	0.9	0.5	0	0	0	2.6	0	0.040
p.P369S	2	0.2	0	0.8	0	0	0.5	0	0.5	0.7	0.5	0.8	1.2	0.5	6.7	8.6	5.8	5.7	5.8	8.1	0.4	0	1.5	0	0.5	0	2.1	2.9	0.5	4.4	1	2	0.000
p.R408Q	1.7	0.2	0	0.8	0	0	0	0	0.5	0.7	0.5	0.8	1.2	0.5	5.5	8.1	3.9	4.8	5.3	5.6	0.4	0	1.5	0	0.5	0	2	2.9	0.5	3.9	1	2	0.000
p.Q440E	0.7	2.6	2.1	1.6	0.5	6.2	1	4.7	1.4	0.3	0	1.6	0	0	0	0	0	0	0	0	0	0	0	0	0	0	0	0	0	0	0	0	0.005
p.461RQ	0.1	0.5	0	0	0	0.9	1.5	0.6	0	0.1	0	0	0.6	0	0	0	0	0	0	0	0	0	0	0	0	0	0	0	0	0	0	0	0.015
p.V487L	0.1	0.3	0.5	0.8	0.5	0	0	0	0.5	0.1	0	0	0	0.5	0	0	0	0	0	0	0	0	0	0	0	0	0	0	0	0	0	0	0.005
p.I591T	0.4	0	0	0	0	0	0	0	0	0.4	1.1	0.8	0	0	0	0	0	0	0	0	1.8	1.5	2	2.2	0.9	2.3	0.1	0.6	0	0	0	0	0.001

The numbers depict percentages. The populations are: AFR: African‐ ACB: African Caribbeans in Barbados‐ ASW: Americans of African Ancestry in SW USA‐ ESN: Esan in Nigeria‐ GWD: Gambian in Western Divisions in the Gambia‐ LWK: Luhya in Webuye, Kenya‐ MSL: Mende in Sierra Leone‐ YRI: Yoruba in Ibadan, Nigeria‐ AMR: American‐ CLM: Colombians from Medellin, Colombia‐ MXL: Mexican Ancestry from Los Angeles USA‐ PEL: Peruvians from Lima, Peru‐ PUR: Puerto Ricans from Puerto Rico‐ EAS: East Asian‐ CDX: Chinese Dai in Xishuangbanna, China‐ CHB: Han Chinese in Bejing, China‐ CHS: Southern Han Chinese‐ JPT: Japanese in Tokyo, Japan‐ KHV: Kinh in Ho Chi Minh City, Vietnam‐ EUR: European‐ CEU: Utah Residents (CEPH) with Northern and Western Europe ‐ FIN: Finnish in Finland‐ GBR: British in England and Scotland‐ IBS: Iberian Population in Spain‐ TSI: Toscani in Italia‐ SAS: South Asian‐ BEB: Bengali from Bangladesh‐ GIH: Gujarati Indian from Houston, Texas‐ ITU: Indian Telugu from the UK‐ PJL: Punjabi from Lahore, Pakistan‐ STU: Sri Lankan Tamil from the UK.

We also analyzed the presence of homozygous mutations in the 1000 Genome database. We found that all nonclustered mutations identified in the 1000 Genome database were also present in the homozygous genotype form in at least two of the five major populations. Homozygous p.E148Q, p.R202Q, and p.G436R were present in all the five major populations, providing further evidence that these mutations were significantly more prevalent in all populations compared to the DCMs.

## Discussion

In this study we analyzed the mutations that have been reported in the *MEFV* gene from symptomatic individuals and could potentially result in FMF symptoms. We studied the substitution patterns for nucleotides and amino acids to determine the conserved and variable regions in the *MEFV* gene. We also analyzed the *MEFV* mutation frequencies in different populations to determine which mutations do not cluster with DCMs. Although the focus of this study was not to assign pathogenicity to the reported mutations, its outcome could provide some insights. This is important since the overwhelming majority of the *MEFV* mutations in symptomatic individuals reported to the FMF database was through personal communications based on findings in individual cases or families; no original research was published on their disease‐causing potential. Although many of these mutations were from case studies from individuals with FMF symptoms, 16 of them were present with high allele frequencies in many non‐FMF prone populations. Particularly, the mutations of interest were p.R42W, p.L110P, p.E148Q, p.R202Q, p.E230K, p.369PS, and p.R408Q, which have been reported in many FMF patients, yet they were also present in many non‐FMF prone populations and had significant allele frequency differences with the DCMs. In fact, p.E148Q, p.369PS, and p.R408Q are routinely tested in many available assays in the market. The p.R42W, p.L110P, p.R202Q, and E230K mutations are not routinely tested with the usual panels and their role in FMF remains unclear. The same results were obtained when the allele frequencies from the Exome database were used for the cluster analysis. This analysis also indicated that these four mutations were present in many populations in addition to the ones with high FMF incidence, suggesting that these mutations may have lesser contributions to the symptoms in individuals with FMF, especially when present in a compound heterozygous genotype with a DCM from another exon.

The 16 mutations that did not cluster with DCMs and were present in high allele frequencies in non‐FMF prone populations were almost exclusively from exons 1 through 5, the only exception was I591T from exon 9. The significance of this finding was that all reported exon 10 mutations clustered with DCMs and therefore could be considered as contributing to the FMF symptoms as far as their allele frequencies are concerned. This emphasizes the significance of exon 10 in the function of the pyrin protein. In fact, the protein structure of pyrin is characterized by an N‐terminal domain, one or two B‐box type zinc fingers followed by a coiled coil motif, and the main C terminal B30.2 domain, which resides in exon 10 and interacts with caspase‐1 (Meroni and Diez‐Roux 2005). What we explain in the next paragraphs is further evidence that the exon 10 C terminal domain is an important functional site in pyrin and therefore mutations in exon 10 cause more severe FMF symptoms. Originally, the FMF mutations, especially in exon 10, were considered loss of function mutations due to their recessive mode of inheritance (Masters et al. 2009). They also caused severe FMF symptoms since mutations in the B30.2 domain, in exon 10, enhance the interaction with caspase‐1 to modulate the release of IL‐1*β* and increase inflammatory response. In turn, caspase‐1 cleaves the B30.2 domain to create a truncated pyrin protein that further could amplify auto‐inflammatory responses, suggesting a gain of function role for mutations in exon 10 or more specifically in B30.2 domain (Masters et al. 2009). Recently, Dorfleutner and Stehlik elegantly proposed a mechanism for pyrin inflammasome activation and control. They explained that the binding of protein 14‐3‐3 to pyrin creates a guard system and its release from pyrin activates the protein and increases the IL‐1*β* production. The 14‐3‐3 and pyrin protein guard system is due to phosphorylation of pyrin by PKN1 and PKN2 at Ser208 and Ser242, which keeps pyrin inactive (Park et al. 2016). Therefore, a mutation in this position in exon 2, (i.e., p.S242R, c.726C>G, rs104895178, c.726C>A, rs104895197) can cause activation of the IL‐1*β* and the inflammosome, acting as a dominant mutation (Masters et al. 2016), due to its position and function in phosphorylation of pyrin, independent of any exon 10 mutation. FMF mutations in B30.2 domain of exon 10 could also prevent the phosphorylation of pyrin and activate it through blocking of PKN1 and PKN2 phosphorylation and subsequently facilitate the release of guard protein 14‐3‐3 from pyrin (Dorfleutner and Stehlik 2016). Also, truncated pyrin without the B30.2 domain in its active form, as well as, mutations in B30.2 domain, which causes more efficient truncation increase the PKN1‐ and PKN2‐dependent phosphorylation of human pyrin, suggesting that B30.2 domain negatively regulates the PKN phosphorylation of pyrin (Dorfleutner and Stehlik 2016).

The molecular evolution analysis of the *MEFV* gene also revealed many interesting findings. The d*n*/d*s* and RAD/CON ratios indicated that exon 2 allows more substitutions and perhaps is less conserved. Moreover, the highest number of mutations not clustered with the DCMs was in exon 2 (6 out of total 16). Although exon 2 mutations could alter the structure of the protein pyrin, their significance in severity of the FMF symptoms may be indistinct. In fact, rarely any severe FMF symptoms have been reported in cases with only exon 2 mutations, with the exception of p.S242R. We have previously reported likely pathogenic exon 2 mutations such as E167D and T267I, along with at least one exon 10 mutation in samples sequenced in symptomatic patients (Moradian et al. 2010). We are yet to see these mutations occur without an exon 10 mutation in individuals with severe FMF symptoms. Based on pyrin activation mechanisms explained above unless the mutations in exon 2 directly block or diminish the PKN‐dependent phosphorylation of pyrin, they may not be able to cause severe FMF symptoms and may need additional mutations in exon 10. But if they do then they can act as dominant mutations independent of exon 10, which further emphasizes our proposal that the location and not the type of the mutation is most likely to determine the severity of the FMF symptoms.

The amino acid substitution pattern seems not to follow the assumption that radical substitutions could alter the protein structure more considerably, consequently resulting in more severe FMF symptoms. It has been reported that the radical substitutions are more likely to be positively selected than conservative ones (Zhang 2000; Hanada et al. 2006). The three DCMs, which cause the most severe FMF symptoms, seem not to follow this assumption since they were all conservative mutations. On the other hand, the 16 mutations that did not cluster with the DCMs had several radical substitutions in them. One can conclude that in case of FMF mutations the location of the mutation in the gene is more significant than the type of the mutation. This is supported by the fact that all three DCMs are in areas with higher conservative substitutions or more conserved areas. Two of them, that is, p.M680I and p.M694V, are in the first conserved segment between amino acids 680 to 704 with a RAD/CON ratio of 0.5 (4/8); p.V726A is in the second area between amino acids 717 and 743 with no radical substitutions compared to nine conservative ones. Approximately two‐thirds of the DCMs are observed in the vicinity of the predicted peptide‐binding site (Weinert et al. 2009) or the conserved region, suggesting that they will have a direct impact on the function of the protein.

A focus of this study was to evaluate the relative frequency of mutations reported in individuals with FMF symptoms and non‐FMF prone populations using statistical clustering. In doing so we found that 16 mutations, close to 8% of nonsynonymous mutations reported to the FMF database, were also present with similar or higher frequencies in non‐FMF prone populations. A future comprehensive study that would focus on evaluating the contributions of these 16 mutations that did not cluster with the DCMs to the FMF symptoms will be a valuable addition to our knowledge about their function. We also concluded that the type of mutation is less likely to determine the FMF symptoms, rather it was the location of the mutations that was the determining factor.

## Conflict of Interest

The authors declare no conflict of interest.
